# Association of rurality, type of primary caregiver and place of death with end-of-life medical expenditures among the oldest-old population in China

**DOI:** 10.1186/s12939-022-01813-2

**Published:** 2023-01-03

**Authors:** Zhong Li, Peiyin Hung, Kewei Shi, You Fu, Dongfu Qian

**Affiliations:** 1 School of Health Policy and Management, Nanjing Medical University, 101 Longmian Avenue, Nanjing, 211166 Jiangsu China; 2 Institution of Healthy Jiangsu Development, Nanjing Medical University, 101 Longmian Avenue, Nanjing, 211166 Jiangsu China; 3 Center for Global Health, Nanjing Medical University, 101 Longmian Avenue, Nanjing, 211166 Jiangsu China; 4 Department of Health Services Policy and Management, Arnold School of Public Health, University of South Carolina, Columbia, SC USA; 5 Surveillance and Health Equity Science, American Cancer Society, Atlanta, GA USA; 6 Department of Review and Investigation, Nanjing Medical University, 101 Longmian Avenue, Nanjing, 211166 Jiangsu China

**Keywords:** Primary Caregivers, Place of Death, End-of-life Care, Medical Expenditures, China

## Abstract

**Background:**

Understanding whether the type of primary caregiver and end-of-life (EOL) care location are associated with EOL medical expenditures is crucial to inform global debates on policies for efficient and effective EOL care. This study aims to assess trends in the type of primary caregiver and place of death stratified by rural‒urban status among the oldest-old population from 1998–2018 in China. A secondary objective is to determine the associations between rurality, the type of primary caregiver, place of death and EOL medical expenditures.

**Methods:**

A total of 20,149 deaths of people aged 80 years or older were derived from the Chinese Longitudinal Health Longevity Survey (CLHLS). Cochran-Armitage tests and Cuzick’s tests were used to test trends in the type of primary caregiver and place of death over time, respectively. Tobit models were used to estimate the marginal associations of rurality, type of primary caregiver, and place of death with EOL medical expenditures because CLHLS sets 100,000 Chinese yuan (approximately US$15,286) as the upper limit of the outcome variable.

**Results:**

Of the 20,149 oldest-old people, the median age at death was 97 years old, 12,490 (weighted, 58.6%, hereafter) were female, and 8,235 lived in urban areas. From 1998–2018, the prevalence of informal caregivers significantly increased from 94.3% to 96.2%, and home death significantly increased from 86.0% to 89.5%. The proportion of people receiving help from informal caregivers significantly *increased* in urban decedents (16.5%) but *decreased* in rural decedents (-4.0%), while home death rates significantly increased among both urban (15.3%) and rural (1.8%) decedents. In the adjusted models, rural decedents spent less than urban decedents did (marginal difference [95% CI]: $-229 [$-378, $-80]). Those who died in hospitals spent more than those who died at home ($798 [$518, $1077]). No difference in medical expenditures by type of primary caregiver was observed.

**Conclusions:**

Over the past two decades, the increases in informal caregiver utilization and home deaths were unequal, leading to substantially higher EOL medical expenditures among urban decedents and deceased individuals who died at hospitals than among their counterparts who lived in rural areas and died at home.

**Supplementary Information:**

The online version contains supplementary material available at 10.1186/s12939-022-01813-2.

## Background

Health and social care systems for older adults are widely concerning worldwide due to aging populations and skyrocketing health care expenditures [1–4]. The World Health Organization projects a nearly doubled increase in the share of the world’s population aged 60 years or older from 12% in 2015 to 22% in 2050 [5]. In China, the population over 65 years old grew rapidly from 8.9% in 2010 to 13.5% in 2020 and is estimated to reach 30% by 2050 [6, 7]. Many young people in rural China have been migrating to metropolitan areas, leaving many older adults behind in rural areas [8], and a recent decrease in family size and changes in attitudes toward kinship obligations have led to much greater needs for institutional caregiving services [9, 10]. Despite this increasing need, nursing home facilities and health care facilities are unevenly distributed across urban and rural communities, potentially exacerbating substantial rural‒urban disparities in access to long-term care [11]. Caregivers play a significant role in the care of older adults with disabilities or serious illness or those in the end of life (EOL) period [12]. Comprehensive care has been proven to effectively meet the multidimensional needs of individuals with terminal illness and improve quality of life coupled with cost savings [13, 14]. Informal care may also reduce the utilization of health care services and related spending and improve discharge outcomes [15, 16]. Moreover, informal caregivers who engage in decision-making about transitioning to long-term care or hospices may pose unique opportunities to mitigate rising EOL expenditures [17, 18].

In China, patients with lower socioeconomic status (SES) rely more on informal care [19]. The long-term care insurance system has been piloted [20], but the limited capacity of the EOL care system has forced many patients with serious illnesses to seek help from and die in hospitals [21]. Disparities in access to care and SES have led to different patterns of EOL care utilization, expenditures, and places of death [22], which calls for the development of community-based EOL care [23]. Although national policies have been enacted to achieve the goals of healthy aging in China [24], only a few hospitals and nursing facilities have reported providing any hospice services due to workforce shortages and insufficient incentives, which results in a greater need for informal care [22]. The forgone time that results from informal caregiving has changed people’s preferences to health care services rather than informal care by family members [25]. Moreover, continuity of care and financial arrangements for long-term care are inadequate [20]. Increased access to and affordability of medical service have made it easier for patients with serious illness and their families to seek help from hospitals, which might cause the overuse of hospital-based care and aggravate the financial burden for family members and the health care system [26]. A fragmented health care system leads to increased medical expenditures and other negative patient outcomes [27, 28]. Understanding the interplay between residential rurality, the type of caregiver, and the place of death and their association with EOL medical expenditures is critical to inform policy enaction and implementation. However, previous studies on EOL care in China have focused on individuals with cancer and other serious illnesses, especially the aging population [21, 22]. To date, no research has investigated the associations of these three key factors and EOL medical expenditures with a sufficient sample size of the oldest-old cohorts. The purpose of this study was to demonstrate the trend of primary caregiver types and place of death stratified by rural‒urban status among the oldest-old population in China from 1998–2018. A secondary objective was to determine the associations of rurality, the type of primary caregiver, and the place of death with EOL medical expenditures.

## Methods

### Study design and participant recruitment

The primary data were derived from the 1998–2018 Chinese Longitudinal Health Longevity Survey (CLHLS), a nationally representative, longitudinal survey administered by the Peking University Chinese Center for Disease Control and Prevention and the China Mainland Information Group. Via in-home proxy interviews with next of kin, family members or close friends, the CLHLS collected a range of health and health care information, including 1) mental health, cognitive function, social participation, nutritional status, family structure, and SES characteristics and 2) health condition, caregivers and medical expenditures during the last year of life among the deceased oldest-old population aged 80 years old or above [29].

The survey, which incorporates a multilevel, stratified, hierarchical sampling procedure across 23 provinces, is the longest dataset for older adult studies in China. Study samples from each wave were weighted using inverse probability weights to produce nationally representative estimates. For this study, seven waves of follow-up surveys among the deceased oldest-old population were conducted from 1998–2018. However, weight was not applied to respondents aged less than 80 or more than 106 years at their first interviews. Therefore, oldest-old individuals aged less than 80 or more than 106 years at their first interviews were excluded from the current study.

### Measures

The key outcome variable was medical expenditures during the last year of life. Each respondent was asked about the *estimated total medical cost in the last year of the decedent’s life*. Our primary independent variables were the deceased’s primary caregivers during the last year of life and place of death. Primary caregivers were reported by the next of kin in the post-death interview, including 1) spouse; 2) adult child(ren); 3) other family members; 4) social care; 5) friends; 6) nurse; 7) no caregiver; and 8) did not need a caregiver. A previous study defined formal care for older adults as paid care services provided by a health care institution or individuals for someone in need and informal care as unpaid care provided by family members, relatives, friends, and neighbors [30]. In this study, informal care consists of care delivered by a spouse, adult child(ren), other family members, and friends, while formal care includes care provided by social institutions and nurses. Place of death was categorized into home, hospitals, and nursing home facilities.

Based on previous studies, the following factors were included as covariates: 1) sociodemographic and socioeconomic characteristics: age at death, gender, ethnicity, years of schooling, number of children, annual per capita household income, primary income sources before dying, marital status, whether living with a spouse or not [2], and white-collar job before retirement [30]; 2) living arrangement during the last year of life: living alone, living in the nursing home, living with a spouse, living with other family members, and timely medical services [2, 17]; 3) health condition: self-rated health status, any disability in activities of daily living (being incontinent or needing assistance in performing one or more activities of bathing, transferring, dressing, eating and toileting) [31], bedridden before dying, and number of comorbidities [19] 4) health behaviors: exercise, drinking and smoking; 5) residential rurality and census region and year of death.

### Statistical analysis

We first used person-level survey weights in the CLHLS dataset to adjust for sampling selection and potential nonresponse or coverage bias. All expenditures and household income were adjusted to the 2018 level according to the National Consumer Price Index. Second, we examined medical expenditures during the last year of life across different groups with Kruskal–Wallis tests. The Cochran-Armitage test and Cuzick’s test were used to assess trends in the type of primary caregiver and place of death, respectively. Third, given that expenditures of 100,000 Chinese yuan (approximately US$15,286) are the upper limit of the outcome variable, we used Tobit regression models to examine the association of rurality, type of primary caregiver and place of death with EOL medical expenditures. Reported *P* values were 2-sided and considered statistically significant at *p*< 0.05. The analysis was conducted between July 1, 2021, and December 10, 2021. The data were managed and analyzed with Stata version 15.1 (College Station, TX). The Strengthening the Reporting of Observational Studies in Epidemiology (STROBE) reporting guideline for prospective cohort studies was followed to report our findings [32]. This CLHLS study was approved by the institutional review board of Peking University and Duke University (IRB00001052-13,074), and each wave was independent of the different participants recruited. Written/oral informal consent was obtained from the respondents or the next of kin in the baseline and follow-up surveys. The current study was performed in accordance with the Declaration of Helsinki, and the Institutional Review Board of Nanjing Medical University considered the analysis of public and anonymous data to be exempt.

### Sensitivity analysis

We used generalized linear regression models with a gamma distribution and log link function after dropping decedents who spent over 100,000 Chinese yuan during the last year of their life. We then examined the association between types of primary caregivers, place of death and medical expenditures stratified by residential rurality. All models were controlled for the aforementioned covariates.

## Results

**Fig. 1:**
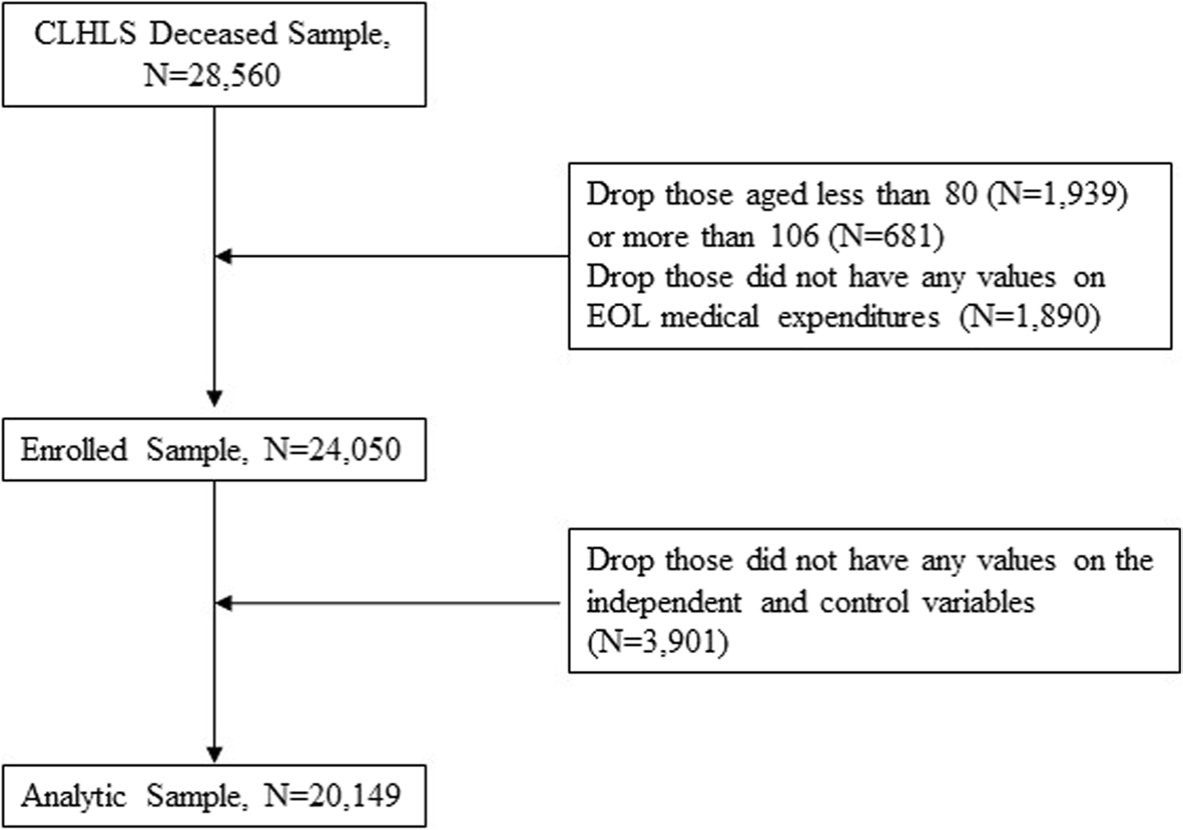
Derivation of Analytic Sample. Note: We first included 3,368 decedents once surveyed in 1998 and died before the 2000 survey, 3,343 once surveyed in 2000 and died before the 2002 survey, 5,874 once surveyed in 2002 and died before the 2015 survey, 5,228 once surveyed in 2005 and died before the 2008 survey, 5,642 once surveyed in 2008 and died before the 2011 survey, 2,879 once surveyed in 2011/2012 and died before the 2014 survey, 2,226 once surveyed in 2014 and died before the 2018 survey. Also, it’s recommended to exclude the respondents who were less than 80 years old if the analysis is focused on the oldest old since they had not reached the oldest old age (80 +) at the survey time. Weight is not applied to those respondents aged less than 80 or more than 106 at their first interviews. CLHLS, Chinese Longitudinal Healthy Longevity Survey

**Fig. 2:**
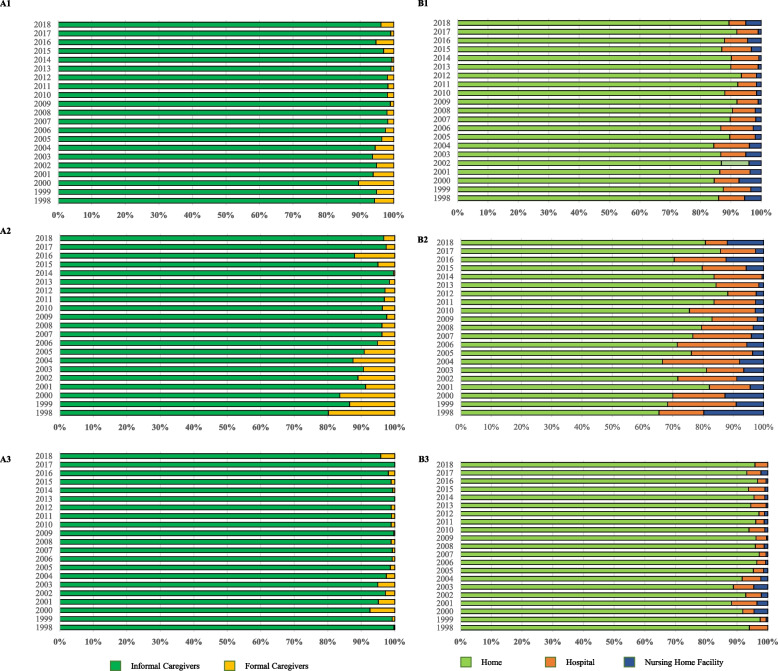
Trends in Type of Primary Caregiver and Place of Death of Sampled Oldest-old by Year of Death. Note: The results were weighted using the age-sex-rural/urban-specific sample weights and reported as column percent. P value for linear trend over the years was determined by the Cochran-Armitage test and Cuzick’s tests, respectively. NHFs, Nursing home facilities. A1, overall trends in prevalence of informal caregivers, 1998–2018 (net change: 1.9%, ptrend < .001); A2, trends in prevalence of informal caregivers among urban deceased, 1998–2018 (net change: 16.5%, ptrend < .001); A3, trends in prevalence of informal caregivers among rural deceased, 1998–2018 (net change: 4.0%, ptrend < .001). B1, overall trends in prevalence of home death, 1998–2018 (net change: 3.5%, ptrend < .001); B2, trends in prevalence of home death among urban deceased, 1998–2018 (net change: 15.3%, ptrend < .001); B3, trends in prevalence of home death among urban deceased, 1998–2018 (net change: 1.8%, ptrend < .001)

Of the 28,560 oldest-old people identified from 1998–2018, we excluded deceased individuals aged less than 80 (*N* = 1,939) or more than 106 years (*N* = 681), 1,890 decedents who did not have any values for EOL medical expenditures, and 3,901 individuals with missing values in any covariates (Fig. 1). The distribution of the oldest-old individuals included by year of death is presented in Supplementary Table 1. The final sample comprised 20,149 oldest-old people (8,235 [41%] urban and 11,914 [59%] rural) with a median age at death of 97 years (Table 1). After weighting using age-sex-rural/urban-specific sample weights, 95.0% of the deceased received help from informal caregivers and 87.5% died at home. Comparisons of weighted sample characteristics by rural‒urban status among the oldest-old population are presented in Table 1. The included deceased had a median EOL medical expenditure of US$ 188. Significant differences in the EOL medical expenditures by residential rurality, type of primary caregivers, place of death and other characteristics are presented in Supplementary Table 2. Figure 2 depicts overall (A1 and B1) trends in the type of primary caregiver and place of death among oldest-old deceased in China and trends by urban (A2 and B2) and rural status (A3 and B3). The prevalence of informal caregivers significantly increased from 94.3% in 1998 to 96.2% in 2018 (*p*_trend_ < 0.001), and the prevalence of home death significantly increased from 86.0% in 1998 to 89.5% in 2018 (*p*_trend_ < 0.001). After stratification by residential rurality, the prevalence of informal caregivers among urban deceased increased from 80.2% in 1998 to 96.7% in 2018, while the prevalence of informal caregivers among rural deceased decreased significantly from 99.8% in 1998 to 95.8% in 2018 (*p*_trend_ < 0.001). The prevalence of home death among urban deceased increased from 65.5% in 1998 to 80.8% in 2018 (*p*_trend_ < 0.001), and the prevalence of home death among rural deceased increased from 94.0% in 1998 to 95.8% in 2018 (*p*_trend_ < 0.001). Detailed information on changes in the patterns of primary caregiver types and place of death are presented in Supplementary Table 3.

**Table 1:** Weighted Sample Characteristics among Oldest-old, 1998–2018

Characteristic	All decedents (*N* = 20,149)	Urban (*N* = 8,235)	Rural (*N* = 11,914)	chi^2^	*P* value
**Informal Caregivers**	19,436 (95.0)	7,724 (90.6)	11,712 (97.2)	35.6	** < .001**
***Place of death***				51.6	** < .001**
Home	18,285 (87.5)	6,820 (75.8)	11,465 (93.2)		
Hospital	1,389 (8.9)	1,086 (17.2)	303 (4.8)		
Nursing home facilities	475 (3.6)	329 (7.0)	146 (2.0)		
**Age at death**	97 (91,102)	96 (91,102)	97 (92,102)	38.3	** < .001**
80–89	3,757 (77.8)	1,666 (75.7)	2,091 (78.9)	10.2	**0.001**
90–99	8,216 (21.4)	3,420 (23.2)	4,796 (20.4)		
≥ 100	8,176 (0.8)	3,149 (1.1)	5,027 (0.7)		
**Gender**				0.3	0.59
Female	12,490 (58.6)	4,956 (58.0)	7,534 (59.0)		
**Ethnicity**				6.2	**0.01**
Han	18,752 (92.4)	7,777 (93.9)	10,975 (91.6)		
**Being married during the last year of life**	2,418 (20.4)	1,106 (24.1)	1,312 (18.6)	14.0	** < .001**
**Years of schooling**				39.7	** < .001**
0	14,738 (67.5)	5,496 (59.6)	9,242 (71.3)		
1–6	4,488 (27.2)	2,158 (31.0)	2,330 (25.4)		
≥ 7	923 (5.3)	581 (9.4)	342 (3.3)		
**Number of children**				4.7	**0.003**
0–2	4,145 (20.4)	1,887 (24.1)	2,258 (18.6)		
3–4	5,849 (29.0)	2,303 (27.0)	3,546 (29.9)		
5–6	5,792 (29.0)	2,293 (27.8)	3,499 (29.6)		
≥ 7	4,363 (21.6)	1,752 (21.1)	2,611 (21.9)		
**Per capita household income annually**				46.0	** < .001**
< 391	4,985 (35.4)	1,551 (25.6)	3,434 (40.1)		
391–942	5,159 (30.5)	1,950 (28.8)	3,209 (31.2)		
942–3,060	4,995 (20.1)	2,170 (24.8)	2,825 (17.9)		
> 3,060	5,010 (14.0)	2,564 (20.8)	2,446 (10.8)		
**Main financial source**					
**Retirement wage**	1,977 (11.2)	1,626 (25.7)	351 (4.1)	241.7	** < .001**
**Family**	16,845 (82.4)	5,965 (65.0)	10,880 (91.0)	281.9	** < .001**
**White-collar jobs before retirement**	816 (4.7)	596 (9.5)	220 (2.4)	83.0	** < .001**
***Living arrangement***					
**living alone**	2,007 (11.3)	735 (10.3)	1,272 (11.8)	2.0	0.16
**living in the nursing home**	486 (4.5)	344 (8.4)	142 (2.5)	31.1	** < .001**
**living with spouse only**	1,940 (12.6)	861 (15.5)	1,079 (11.2)	14.3	** < .001**
**living with other family members**	15,373 (70.3)	6,117 (64.2)	9,256 (73.3)	29.0	** < .001**
**timely medical services**				4.7	**0.01**
Yes	15,014 (81.6)	6,310 (83.8)	8,704 (80.5)		
No	1,322 (8.2)	572 (8.1)	750 (8.2)		
Was not ill	3,760 (10.2)	1,334 (8.1)	2,426 (11.3)		
***Health Condition***					
**Self-rated health status**				0.9	0.43
Very good or good	7,415 (41.2)	3,105 (40.3)	4,310 (41.6)		
So so	6,127 (32.4)	2,561 (34.2)	3,566 (31.6)		
Bad or very bad	3,152 (19.7)	1,215 (18.9)	1,937 (20.2)		
Not able to answer	3,455 (6.7)	1,354 (6.6)	2,101 (6.8)		
**Any disability in ADLs**	3,581 (25.6)	1,316 (21.7)	2,265 (27.5)	12.3	** < .001**
**Bedridden before dying**	14,947 (27.1)	6,143 (25.8)	8,804 (27.8)	1.5	0.23
**No of comorbidities**				12.9	** < .001**
0	7,248 (33.1)	2,502 (27.7)	4,746 (35.6)		
1	6,466 (32.9)	2,538 (31.8)	3,928 (33.5)		
2	2,921 (15.1)	1,307 (16.7)	1,614 (14.4)		
≥ 3	3,514 (18.9)	1,888 (23.8)	1,626 (16.5)		
***Health Behavior***^a^					
**Physical exercise**	3,920 (24.6)	2,011 (31.4)	1,909 (21.3)	38.6	** < .001**
**Smoking**	2,832 (18.6)	1,065 (15.9)	1,767 (20.0)	9.0	**0.003**
**Drinking**	3,233 (15.9)	1,242 (14.4)	1,991 (16.7)	3.4	0.07
**Region**				25.1	** < .001**
Eastern	7,714 (35.6)	3,347 (36.1)	4,367 (35.4)		
Central	4,688 (24.5)	1,430 (18.0)	3,258 (27.7)		
Western	6,161 (31.4)	2,471 (32.2)	3,690 (31.0)		
Northeast	1,586 (8.5)	987 (13.7)	599 (5.9)		

Note: Values were represented as No. (percentages) unless otherwise indicated. No, was calculated from study samples (unweighted). Percentages were calculated using the age-sex-rural/urban-specific sample weights; a, self-reported in the previous survey. ADLs, activities of daily living. All the currency was presented in the US dollars

Adjusted models showed that deceased individuals who received informal care no longer differed from deceased individuals who received formal care in terms of EOL medical expenditure (marginal differences [95% confidence interval]: $278 [$-443 to $1000]; Table 2). Rural deceased had less medical expenditures than urban deceased did ($-229 [$-378 to $-80]). Compared to those who died at home, individuals who died at hospitals had greater medical expenditures ($798 [$518 to $1,077]), while individuals who died at nursing home facilities did not ($-440 [$-1,055 to $176]). Individuals older than 90 years old ($-180 [$-274, $-86]) and centenarians ($-424 [$-546, $-302]) spent less than deceased individuals younger than 90 years old. Individuals from the most affluent families ($726 [$452, $999]) or who received retirement wages as their main financial source ($551 [$ 120, $902]) had greater medical expenditures than their counterparts did. Individuals who did not have timely medical services ($-335 [$-446, $-223]) or who were not ill ($-317 [$-451, $-183]) spent less than those who died with timely medical services. Individuals with any disabilities in activities of daily living ($313 [$178, $448]) or those suffering from two or more chronic diseases had greater medical expenditures than their counterparts did.

**Table 2:** Marginal differences of included characteristics on medical expenditure during the last year of life

Characteristics	Dy/dx	95% CI		*P* value
**Rurality (Ref: Urban)**				
Rural	-229	-378	-80	**0.003**
**Primary Caregivers (Ref: Informal)**				
Formal	278	-443	1,000	0.45
**Place of death (Ref: Home)**				
Hospital	798	518	1,077	** < .001**
Nursing home facilities	-440	-1,055	176	0.161
**Age at death (Ref: 80–89)**				
90–99	-180	-274	-86	** < .001**
≥ 100	-424	-546	-302	** < .001**
**Per capita household income annually (Ref: < 391)**				
391–942	4	-124	132	0.95
942–3,060	206	-26	438	0.08
> 3,060	726	452	999	** < .001**
**Main financial source before dying**				
**Retirement wage (Ref: No)**	511	120	902	**0.01**
**Timely medical services (Ref: Yes)**				
No	-335	-446	-223	** < .001**
Was not ill	-317	-451	-183	** < .001**
***Health Condition***				
**Disabilities in ADLs (Ref: No)**	313	178	448	** < .001**
**Number of Comorbidities (Ref: 0)**				
1	-4	-127	120	0.95
2	177	25	330	**0.02**
≥ 3	224	15	433	**0.04**
**Region (Ref: Eastern)**				
Central	-264	-425	-103	**0.001**
Western	-282	-436	-128	** < .001**
Northeast	-154	-419	111	0.26

Note: *Dy/dx* Average marginal effect, *CI* Confidence interval, *ADLs* activities of daily living. Only results of predictors of statistically significance were presented. Results of associations between gender, ethnicity, marital status, years of schooling, number of children even born, family support as main financial source, once having white-collar jobs before retirement, living arrangement during the last year of life, self-rated health status, health behaviors and end-of-life medical expenditures were not presented. All the currency was presented in the US dollar

The results of the generalized linear regression model remain consistent with our main findings in both direction and significance (Supplementary Table 4). After stratification by urban and rural status, the results remain similar to the main findings. Urban deceased who died at hospital spent more than those who died at home ($967 [$578 to $1,357]). Rural deceased individuals who died at a hospital spent more than those who died at home ($594 [$243 to $994]), which is far less than the results of our main findings (Supplementary Tables 5 and  6 in the Supplement).

## Discussion

To the best of our knowledge, this study is the first to explore trends in the type of primary caregiver and place of death as well as their associations with EOL expenditures. This study provides vital information about the magnitude of the problem to enrich current research on EOL care, inform policy efforts to improve access to home and informal care, and reduce substantial disparities in EOL medical expenditures. Our data indicate that more than 95% of oldest-old individuals received help from informal caregivers, and nearly 90% of them died at home as of 2018. Moreover, urban deceased spent much more than rural deceased did, and those who died in hospitals spent more than those who died at home. This suggests that persistent challenges remain for increasing access to out-of-hospital EOL care services among oldest-old individuals in China.

Currently, formal care is not routinely used to deliver health and social care among oldest-old individuals. Nursing home facilities are far less likely to be the place of death in China, with only approximately 5% of deaths occurring in nursing home facilities in 2018. Although formal care has long been advocated to improve access to care for older adults and to improve continuity of care between health care and social care providers, its use by oldest-old individuals is limited. Rates of informal care utilization among the oldest-old increased from 1998 to 2018, especially the prevalence of informal care among urban deceased. This result is similar to the findings of one study conducted in the US that showed that rates of informal home care use among older adults with disabilities increased from 2004 to 2016 despite 36.9% of formal home care use in 2016 [2]. However, many families face barriers to engaging in formal care, especially due to persistent cultural obstacles [10] and longstanding disconnects between hospitals and primary care facilities [33]. Differences in care continuity mean sizable differences in the utilization of services and costs [34]. More importantly, the prevalence of home death increased from 86.0% in 1998 to 89.5% in 2018, and similar trends were observed among the urban and rural deceased, suggesting that more intensive education, training and support for informal caregivers is warranted [33, 35–37]. Informal caregiving for elderly people with disabilities has been commonly used to provide services similar to those provided to paid formal caregivers in China [38]. However, with limited community-based care, family caregivers are unaware of what they do not know or should do. To meet the needs of oldest-old individuals during the last year of life, informal caregivers must receive expanded access to supportive services [39].

We found that urban‒rural disparities in the EOL medical expenditure were especially pronounced among those who died in the hospitals. Different affordability and access to care, especially hospital-based care, mean disproportionate utilization of inpatient services among individuals with severe diseases. Moreover, the medical expenditures of individuals who primarily received formal care did not differ from those of deceased individuals who primarily received informal care. This finding may indicate potential opportunities to train formal caregivers to provide services comparable to services in hospital settings without increased medical expenditures [40]. In addition, unintentional incentives for caregivers to provide a longer length of stay might increase medical expenditures [41]. Therefore, continuity of care between hospitals and primary care facilities for oldest-old individuals who are discharged from hospitals is a major issue.

Given that EOL discussion is associated with less aggressive care near death and earlier hospice referrals [42, 43], it is concerning that EOL medical expenditures varied significantly by the age when death occurred, particularly in terms of greater expenditures among deceased individuals aged between 80 and 89 years. This study is similar to findings of a previous study showing that Medicare expenditures and the aggressiveness of medical care decreased in the last year of life and decreased with age [44]. Previous studies also showed that individuals with multimorbidity had substantially greater medical expenditures [45], and individuals living in communities with a high SES were almost twice as likely to die in homes than individuals from low-SES communities [46]. Patients with advanced illnesses and their families often decide to use life-prolonging treatments [33]. Given that awareness of the terminal nature of life may reduce unnecessary costs, educating caregivers may be an important part of advance care planning and related policy-making [47]. Because formal caregivers are not associated with EOL medical expenditures, purposive policies are urgently needed to address the increasing need for informal caregivers. This is particularly important for those who died at hospitals, urban and affluent deceased, and individuals with any disability in daily activities who are bedridden.

Our findings should be interpreted with the following potential limitations. Because the CLHLS data do not provide longitudinal weights, the application of survey weights could cause biased estimates because of nonrandom losses of follow-up. First, we were limited to decedents without any missing values in any covariates. The excluded deceased were more likely to be cared for by informal care and to die at home, have lower income, receive financial support from their families, live alone, receive timely medical services, report having any disabilities in activities of daily living and being bedridden, and smoke and drink in the last year of life (Supplementary Table 7). Therefore, our results may not be generalizable to all oldest-old individuals. We were also not able to identify the cause of death. The type of insurance in which deceased individuals were enrolled also has a large proportion of missing values. We therefore took main financial support to represent the affordability of medical services. Although we used the number of chronic diseases as covariates, subgroups such as individuals with cancer or dementia were limited to capturing meaningful differences. Future studies should consider these groups with a greater need for EOL care. EOL medical expenditure may also be affected by the utilization of health services; however, in this dataset, there are no specific variables that indicate the number of inpatient and outpatient utilizations during the last year of life.

## Conclusion

This study provides vital information about trends in the type of primary caregiver and place of death and their association with medical expenditures among the oldest-old population in China during the past two decades. The prevalence of informal caregivers significantly increased from 94.3% in 1998 to 96.2% in 2018, and the prevalence of home death significantly increased from 86.0% from 1996 to 89.5% in 2018. Significant regional and rural‒urban disparities in EOL medical expenditures remain. Our study also found that place of death, SES and health conditions were significant predictors of EOL medical expenditures. Deceased from urban areas and those who died in hospitals, had higher SES, and had worse health conditions spent more than their counterparts. Given the well-documented benefits of informal and home care, policy-makers and health care professionals need to pay particular attention to these deceased and to incorporate their illness trajectory and goals of care to reduce disparities in EOL medical expenditures.

## Supplementary Information

**Additional file 1:** **Supplementary Table 1.** Distribution of sampled oldest-old by year of death. **Supplementary Table 2.** Medical expenditures during the last year of life by sample characteristics. **Supplementary Table 3.** trends in type of primary caregiver and place of death of sampled oldest-old by year of death. **Supplementary Table 4.** Results of sensitivity analysis. **Supplementary Table 5.** Marginal differences of included characteristics on medical expenditure during the last year of life among urban deceased. **Supplementary Table 6.** Marginal differences of included characteristics on medical expenditure during the last year of life among rural deceased. **Supplementary Table 7.** Comparison of individual characteristics between deceased reported and not reported medical expenditure during the last year of life.

## Data Availability

All the data were available from Peking University Open Research Data. Center for Healthy Aging and Development Studies, 2020, "The Chinese Longitudinal Healthy Longevity Survey (CLHLS)-Longitudinal Data (1998–2018) ", https://doi.org/10.18170/DVN/WBO7LK, Peking University Open Research Data Platform, V2.

## Abbreviations

EOL: End of Life
CLHLS: Chinese Longitudinal Health Longevity Survey
US: United States
SES: Socio-economics status
STROBE: The Strengthening the Reporting of Observational Studies in Epidemiology

## References

[1] Zeng Y, Feng Q, Hesketh T, Christensen K, Vaupel JW (2017). Survival, disabilities in activities of daily living, and physical and cognitive functioning among the oldest-old in China: a cohort study. Lancet.

[2] Van Houtven CH, Konetzka RT, Taggert E, Coe NB (2020). Informal And Formal Home Care For Older Adults With Disabilities Increased, 2004–16: Study examines changes in the rates of informal home care use among older adults with disabilities 2004 to 2016. Health Aff.

[3] Fiest KM, McIntosh CJ, Demiantschuk D, Leigh JP, Stelfox HT (2018). Translating evidence to patient care through caregivers: a systematic review of caregiver-mediated interventions. BMC Med.

[4] Desa U N. World population prospects 2019: Highlights[J]. New York (US): United Nations Department for Economic and Social Affairs. 2019;11(1):125.

[5] World Health Organization. Ageing and Health [Internet]. Available from: https://www.who.int/news-room/fact-sheets/detail/ageing-and-health. Accessed 30 Dec 2022.

[6] National Bureau of Statistics. Bulletin of the Seventh National Census[1](No.5)—Population Characteristics by Age [Internet]. Available from: http://www.stats.gov.cn/tjsj/zxfb/202105/t20210510_1817181.html. Accessed 12 Dec 2021.

[7] Luo Y, Su B, Zheng X (2021). Trends and challenges for population and health during population aging—China, 2015–2050[J]. China CDC Weekly.

[8] National Health Commission, Center for Health Statistics and Informantion (2020). An Anlysis Report of National Health Services Surveys in China, 2018. Healthcare need and self-rated status.

[9] Liao J, Wu B, Ni P, Mao J (2019). Advance directive preferences among terminally ill older patients and its facilitators and barriers in China: a scoping review. J Am Med Dir Assoc.

[10] Cheng G, Chen C. End-of-Life Needs of Dying Patients and Their Families in Mainland China: A Systematic Review. Omega: J. Death Dying, 2021:0030222821997340. 10.1177/0030222821997340. 10.1177/0030222821997340 33626990

[11] Fang H, Chen J, Rizzo JA (2009). Explaining urban-rural health disparities in China. Med Care.

[12] Rabow MW, Hauser JM, Adams J (2004). Supporting family caregivers at the end of life: They don't know what they don't know. JAMA.

[13] Wright AA, Keating NL, Balboni TA, Matulonis UA, Block SD, Prigerson HG (2010). Place of death: correlations with quality of life of patients with cancer and predictors of bereaved caregivers' mental health. J Clin Oncol.

[14] Carlson MDA, Herrin J, Du Q (2010). Impact of hospice disenrollment on health care use and medicare expenditures for patients with cancer. J Clin Oncol.

[15] Van Houtven CH, Norton EC (2008). Informal care and Medicare expenditures: Testing for heterogeneous treatment effects. J Health Econ.

[16] Simning A, Orth J, Wang J (2020). Skilled nursing facility patients discharged to home health agency services spend more days at home. J Am Geriatr Soc.

[17] Hung P, Hsu SH, Wang SY (2020). Associations between end-of-life expenditures and hospice stay length vary by clinical condition and expenditure duration. Value Health.

[18] Lackraj D, Kavalieratos D, Murali KP, Lu Y, Hua M (2021). Implementation of Specialist Palliative Care and Outcomes for Hospitalized Patients with Dementia. J Am Geriatr Soc.

[19] Johnson MJ, Allgar V, Chen H, Dunn L, Macleod U, Currow DC (2018). The complex relationship between household income of family caregivers, access to palliative care services and place of death: a national household population survey. Palliat Med.

[20] Feng Z, Glinskaya E, Chen H (2020). Long-term care system for older adults in China: policy landscape, challenges, and future prospects. Lancet.

[21] Li Z, Jiang S, Xu C (2020). Determinants of place of death for end-stage cancer patients: evidence from China. Int J Qual Health Care.

[22] Li Z, Hung P, He R (2020). Disparities in end-of-life care, expenditures, and place of death by health insurance among cancer patients in China: a population-based, retrospective study. BMC Public Health.

[23] Zhou J, Walker A (2016). The need for community care among older people in China. Ageing Soc.

[24] Ye P, Jin Y, Er Y (2021). A Scoping Review of National Policies for Healthy Ageing in Mainland China from 2016 to 2020. Lancet Reg Health West Pac.

[25] Van Houtven CH, Coe NB, Skira MM (2013). The effect of informal care on work and wages[J]. J Health Econ.

[26] Costa-Font J, Courbage C (2015). Crowding out of long-term care insurance: Evidence from European expectations data. Health Econ.

[27] Hung P, Cramer LD, Pollack CE, Gross CP, Wang SY (2021). Primary care physician continuity, survival, and end-of-life care intensity. Health Serv Res.

[28] Augustine MR, Nelson KM, Fihn SD, Wong ES (2019). Patient-reported access in the patient-centered medical home and avoidable hospitalizations: an observational analysis of the Veterans Health Administration. J Gen Intern Med.

[29] Center for Healthy Aging and Development Studies, 2020, "The Chinese Longitudinal Healthy Longevity Survey (CLHLS)-Longitudinal Data(1998–2018)", 10.18170/DVN/WBO7LK, Peking University Open Research Data Platform, V2 [Internet]. Accessed 30 Dec 2022.

[30] Gu, Danan, and Matthew E. Dupre. *Encyclopedia of gerontology and population aging*. Cham: Springer International Publishing, 2020. Chapter: Formal and Informal Care. Available from: 10.1007/978-3-319-69892-2_847-1. Accessed 30 Dec 2022.

[31] Liu Z, Han L, Wang X, Feng Q, Gill TM (2018). Disability prior to death among the oldest-old in China. J Gerontol A Biol Sci Med Sci.

[32] von Elm E, Altman DG, Egger M, Pocock SJ, Gøtzsche PC, Vandenbroucke JP (2007). Strengthening the reporting of observational studies in epidemiology (STROBE) statement: guidelines for reporting observational studies. BMJ.

[33] Chung H, Harding R, Guo P (2021). Palliative care in the greater China region: a systematic review of needs, models, and outcomes. J Pain Symptom Manag.

[34] Hussey PS, Schneider EC, Rudin RS, Fox DS, Lai J, Pollack CE (2014). Continuity and the costs of care for chronic disease. JAMA Intern Med.

[35] Wright AA, Zhang B, Ray A (2008). Associations between end-of-life discussions, patient mental health, medical care near death, and caregiver bereavement adjustment. JAMA.

[36] Pottie CG, Burch KA, Montross Thomas LP (2014). Informal caregiving of hospice patients. J Palliat Med.

[37] Ornstein KA, Aldridge MD, Garrido MM (2015). Association between hospice use and depressive symptoms in surviving spouses. JAMA Intern Med.

[38] Zhu S, Zhu H, Zhang X (2021). Care needs of dying patients and their family caregivers in hospice and palliative care in mainland China: a meta-synthesis of qualitative and quantitative studies. BMJ Open.

[39] Lindqvist O, Tishelman C, Hagelin CL (2012). Complexity in non-pharmacological caregiving activities at the end of life: an international qualitative study. PLoS Med.

[40] Davies JM, Maddocks M, Chua KC, Demakakos P, Sleeman KE, Murtagh FE (2021). Socioeconomic position and use of hospital-based care towards the end of life: a mediation analysis using the English Longitudinal Study of Ageing. Lancet Public Health.

[41] Singh S, Lum HD, Kutner J (2021). The patient-driven payment model: A missed opportunity for patient-centered cancer care. J Am Geriatr Soc.

[42] Nicholas LH, Bynum JP, Iwashyna TJ, Weir DR, Langa KM (2014). Advance directives and nursing home stays associated with less aggressive end-of-life care for patients with severe dementia. Health Aff.

[43] Mor V, Wagner TH, Levy C (2019). Association of expanded VA hospice care with aggressive care and cost for veterans with advanced lung cancer. JAMA Oncol.

[44] Levinsky NG, Yu W, Ash A (2001). Influence of age on Medicare expenditures and medical care in the last year of life. JAMA.

[45] Ankuda CK, Maust DT, Kabeto MU (2017). Association between spousal caregiver well-being and care recipient healthcare expenditures. J Am Geriatr Soc.

[46] Houttekier D, Cohen J, Bilsen J, Addington-Hall J, Onwuteaka-Philipsen B, Deliens L (2010). Place of death in metropolitan regions: metropolitan versus non-metropolitan variation in place of death in Belgium The Netherlands and England. Health Place.

[47] Van der Steen JT, Onwuteaka-Philipsen BD, Knol DL, Ribbe MW, Deliens L (2013). Caregivers’ understanding of dementia predicts patients’ comfort at death: a prospective observational study. BMC Med.

