# Supplementary material for: Association of rurality, type of primary caregiver and place of death with end-of-life medical expenditures among the oldest-old population in China
Source: Int J Equity Health. 2023 Jan 3;22:1. doi: 10.1186/s12939-022-01813-2 (PMC9809123; doi:10.1186/s12939-022-01813-2)
Supplement: Supplementary file 1 — Additional file 1: Supplementary Table 1. Distribution of sampled oldest-old by year of death. Supplementary Table 2. Medical expenditures during the last year of life by sample characteristics. Supplementary Table 3. trends in type of primary caregiver and place of death of sampled oldest-old by year of death. Supplementary Table 4. Results of sensitivity analysis. Supplementary Table 5. Marginal differences of included characteristics on medical expenditure during the last year of life among urban deceased. Supplementary Table 6. Marginal differences of included characteristics on medical expenditure during the last year of life among rural deceased. Supplementary Table 7. Comparison of individual characteristics between deceased reported and not reported medical expenditure during the last year of life. [file 12939_2022_1813_MOESM1_ESM.doc]

# **Supplement**

# Supplementary Table 1. Distribution of Sampled Oldest-old by Year of Death

# Supplementary Table 2. Medical Expenditures during the Last Year of Life by Sample Characteristics

# Supplementary Table 3. Trends in Type of Primary Caregiver and Place of Death of Sampled Oldest-old by Year of Death

# Supplementary Table 4. Results of Sensitivity Analysis

# Supplementary Table 5. Marginal Differences of Included Characteristics on Medical Expenditure during the Last Year of Life among Urban Deceased

# Supplementary Table 6. Marginal Differences of Included Characteristics on Medical Expenditure during the Last Year of Life among Rural Deceased

# Supplementary Table 7. Comparison of Individual Characteristics between Deceased Reported and not Reported Medical Expenditure during the Last Year of Life

# **Supplementary Table 1. Distribution of Sampled Oldest-old by Year of Death**

| **Year of death** | **Frequency** | **%** | **Cumulative %** |
| --- | --- | --- | --- |
| 1998 | 587 | 2.9 | 2.9 |
| 1999 | 1,272 | 6.3 | 9.2 |
| 2000 | 1,118 | 5.6 | 14.8 |
| 2001 | 1,621 | 8.1 | 22.8 |
| 2002 | 1,092 | 5.4 | 28.2 |
| 2003 | 1,619 | 8.0 | 36.3 |
| 2004 | 1,473 | 7.3 | 43.6 |
| 2005 | 972 | 4.8 | 48.4 |
| 2006 | 1,385 | 6.9 | 55.3 |
| 2007 | 1,285 | 6.4 | 61.7 |
| 2008 | 1,029 | 5.1 | 66.8 |
| 2009 | 1,411 | 7.0 | 73.8 |
| 2010 | 1,339 | 6.7 | 80.4 |
| 2011 | 904 | 4.5 | 84.9 |
| 2012 | 793 | 3.9 | 88.8 |
| 2013 | 651 | 3.2 | 92.1 |
| 2014 | 487 | 2.4 | 94.5 |
| 2015 | 370 | 1.8 | 96.3 |
| 2016 | 350 | 1.7 | 98.0 |
| 2017 | 292 | 1.5 | 99.5 |
| 2018 | 99 | 0.5 | 100.0 |

# **Supplementary Table 2. Medical Expenditures during the Last Year of Life by Sample Characteristics**

| **Characteristic** | **Medical Expenditures** | **chi^2^** | ***P* value** |
| --- | --- | --- | --- |
| **Overall** | 188 (43, 639) |  |  |
| **Rurality** |  | 340.2 | **< .001** |
| Urban | 245 (61, 907) |  |  |
| Rural | 160 (39, 490) |  |  |
| ***Primary Caregivers*** |  | 2.3 | **0.13** |
| Informal | 188 (43, 639) |  |  |
| Formal | 212 (43, 742) |  |  |
| ***Place of death*** |  | 1100.1 | **< .001** |
| Home | 173 (41, 553) |  |  |
| Nursing home facilities | 178 (39, 592) |  |  |
| Hospital | 987 (356, 2575) |  |  |
| **Age at death** |  | 632.8 | **< .001** |
| 80-89 | 377 (97, 1067) |  |  |
| 90-99 | 197 (43, 644) |  |  |
| ≥100 | 142 (32, 426) |  |  |
| **Gender** |  | 187.8 | **< .001** |
| Male | 215 (57, 851) |  |  |
| Female | 171 (40, 537) |  |  |
| **Ethnicity** |  | 153.8 | **< .001** |
| Han | 197 (43, 644) |  |  |
| Minority | 90 (10, 395) |  |  |
| **Being married** |  | 367.0 | **< .001** |
| Yes | 408 (99, 1384) |  |  |
| No | 173 (41, 592) |  |  |
| **Years of schooling** |  | 308.4 | **< .001** |
| 0 | 172 (41, 537) |  |  |
| 1-6 | 237 (60, 889) |  |  |
| ≥ 7 | 429 (102, 1319) |  |  |
| **Number of children even born** |  | 14.1 | **0.01** |
| 0-2 | 179 (43, 612) |  |  |
| 3-4 | 179 (42, 613) |  |  |
| 5-6 | 197 (43, 717) |  |  |
| ≥ 7 | 188 (43, 639) |  |  |
| **Per capita household income annually** |  | 445.6 | **< .001** |
| < 391 | 106 (21, 362) |  |  |
| 391-942 | 156 (42, 429) |  |  |
| 942-3,060 | 212 (57, 729) |  |  |
| > 3,060 | 395 (89, 1392) |  |  |
| **Main financial source** |  |  |  |
| **Retirement wage** |  | 1554.3 | **< .001** |
| Yes | 756 (197, 2129) |  |  |
| No | 172 (41, 537) |  |  |
| **Family** |  | 1554.3 | **< .001** |
| Yes | 173 (41, 537) |  |  |
| No | 401 (87, 1459) |  |  |
| **White-collar jobs before retirement** |  | 369.2 | **< .001** |
| Yes | 625 (156, 1905) |  |  |
| No | 179 (42, 612) |  |  |
| ***Living arrangement*** |  |  |  |
| **living alone** |  | 16.0 | **0.03** |
| Yes | 188 (43, 636) |  |  |
| No | 188 (43, 639) |  |  |
| **living in the nursing home** |  | 191.7 | **< .001** |
| Yes | 165 (21, 636) |  |  |
| No | 188 (43, 639) |  |  |
| **living with spouse only** |  | 8.5 | **< .001** |
| Yes | 377 (82, 1347) |  |  |
| No | 179 (42, 601) |  |  |
| **living with other family members** |  | 31.0 | **< .001** |
| Yes | 179 (43, 601) |  |  |
| No | 214 (47, 849) |  |  |
| **timely medical services** |  | 55.9 | **< .001** |
| Yes | 241 (80, 802) |  |  |
| No | 67 (0, 279) |  |  |
| Was not ill | 48 (0, 204) |  |  |
| ***Health Condition*** |  |  |  |
| **Self-rated health status** |  | 16.9 | **< .001** |
| Very good or good | 188 (42, 639) |  |  |
| So so | 197 (43, 645) |  |  |
| Bad or very bad | 214 (61, 756) |  |  |
| Not able to answer | 160 (35, 520) |  |  |
| **Any disability in ADLs** |  | 30.8 | **< .001** |
| Yes | 213 (64, 744) |  |  |
| No | 64 (0, 215) |  |  |
| **Bedridden before dying** |  | 1.3 | **0.25** |
| Yes | 214 (64, 754) |  |  |
| No | 88 (11, 347) |  |  |
| **No of Comorbidities** |  | 390.5 | **< .001** |
| 0 | 154 (31, 519) |  |  |
| 1 | 175 (42, 565) |  |  |
| 2 | 213 (64, 802) |  |  |
| ≥ 3 | 319 (88, 987) |  |  |
| ***Health Behavior*** ^a^ |  |  |  |
| **Physical exercise** |  | 224.9 | **< .001** |
| Yes | 215 (57, 878) |  |  |
| No | 178 (42, 601) |  |  |
| **Smoking** |  | 16.0 | **0.58** |
| Yes | 188 (42, 658) |  |  |
| No | 188 (43, 639) |  |  |
| **Drinking** |  | 9.3 | **0.03** |
| Yes | 178 (41, 636) |  |  |
| No | 191 (43, 639) |  |  |
| **Region** |  | 535.8 | **< .001** |
| Eastern | 213 (53, 781) |  |  |
| Central | 171 (42, 494) |  |  |
| Western | 163 (40, 576) |  |  |
| Northeast | 213 (48, 816) |  |  |

Note: Values were presented as Median (p25, p75) unless otherwise indicated; a, self-reported in the previous survey. All the currency was presented in the US dollar.

# **Supplementary Table 3. Trends in Type of** Primary Caregiver and Place of Death of Sampled Oldest-old by Year of Death

| **Year of death** | **Type of Primary caregiver** | | | | | | **Place of death** | | | | | | | | |
| --- | --- | --- | --- | --- | --- | --- | --- | --- | --- | --- | --- | --- | --- | --- | --- |
|  | **Overall** | | **Urban** | | **Rural** | | **Overall** | | | **Urban** | | | **Rural** | | |
|  | **Informal** | **Formal** | **Informal** | **Formal** | **Informal** | **Formal** | **Home** | **Hospital** | **NHFs** | **Home** | **Hospital** | **NHFs** | **Home** | **Hospital** | **NHFs** |
| 1998 | 94.3 | 5.7 | 80.2 | 19.8 | 99.8 | 0.2 | 86.0 | 8.5 | 5.5 | 65.5 | 14.8 | 19.7 | 94.0 | 6.0 | 0 |
| 1999 | 94.9 | 5.1 | 86.5 | 13.5 | 99.2 | 0.8 | 87.6 | 9.1 | 3.3 | 68.3 | 22.7 | 9.0 | 97.5 | 2.0 | 0.5 |
| 2000 | 89.5 | 10.5 | 83.6 | 16.4 | 92.6 | 7.4 | 84.5 | 8.2 | 7.3 | 70.1 | 17.3 | 12.6 | 91.9 | 3.6 | 4.5 |
| 2001 | 93.9 | 6.1 | 91.3 | 8.7 | 95.1 | 4.9 | 86.3 | 9.9 | 3.8 | 82.1 | 13.5 | 4.4 | 88.2 | 8.3 | 3.5 |
| 2002 | 94.9 | 5.1 | 89.0 | 11.0 | 97.2 | 2.8 | 87.0 | 9.0 | 4.0 | 71.8 | 19.5 | 8.8 | 92.9 | 5.1 | 2.0 |
| 2003 | 93.7 | 6.3 | 90.6 | 9.4 | 94.9 | 5.1 | 86.7 | 8.2 | 5.1 | 81.2 | 12.4 | 6.4 | 88.8 | 6.6 | 4.6 |
| 2004 | 94.5 | 5.5 | 87.5 | 12.5 | 97.5 | 2.5 | 84.3 | 11.7 | 4.0 | 66.6 | 25.5 | 7.9 | 91.7 | 6.0 | 2.3 |
| 2005 | 96.5 | 3.5 | 90.9 | 9.1 | 98.7 | 1.3 | 89.8 | 8.2 | 2.0 | 76.2 | 20.2 | 3.6 | 95.3 | 3.3 | 1.4 |
| 2006 | 97.5 | 2.5 | 94.8 | 5.2 | 99.2 | 0.8 | 86.7 | 10.7 | 2.6 | 71.5 | 23.0 | 5.5 | 96.4 | 2.9 | 0.7 |
| 2007 | 98.2 | 1.8 | 96.2 | 3.8 | 99.3 | 0.7 | 89.8 | 8.4 | 1.8 | 76.6 | 19.3 | 4.1 | 97.2 | 2.2 | 0.6 |
| 2008 | 98.0 | 2.0 | 96.2 | 3.8 | 98.9 | 1.1 | 90.6 | 7.4 | 2.0 | 79.5 | 17.0 | 3.5 | 95.9 | 2.8 | 1.3 |
| 2009 | 99.0 | 1.0 | 97.6 | 2.4 | 99.7 | 0.3 | 92.0 | 7.0 | 1.0 | 83.0 | 14.9 | 2.1 | 96.1 | 3.4 | 0.5 |
| 2010 | 98.1 | 1.9 | 96.3 | 3.7 | 98.9 | 1.1 | 88.0 | 10.5 | 1.5 | 74.9 | 21.6 | 2.5 | 93.9 | 5.1 | 1.0 |
| 2011 | 98.3 | 1.7 | 96.9 | 3.1 | 99.0 | 1.0 | 92.3 | 6.1 | 1.6 | 83.9 | 13.8 | 2.3 | 96.2 | 2.7 | 1.1 |
| 2012 | 98.1 | 1.9 | 97.1 | 2.9 | 98.9 | 1.1 | 93.5 | 4.9 | 1.6 | 88.2 | 9.4 | 2.4 | 97.1 | 1.8 | 1.1 |
| 2013 | 99.2 | 0.8 | 98.4 | 1.6 | 100.0 | 0 | 89.9 | 9.0 | 1.1 | 84.3 | 14.1 | 1.6 | 94.5 | 5.0 | 0.5 |
| 2014 | 99.5 | 0.5 | 99.6 | 0.4 | 99.4 | 0.6 | 90.2 | 9.0 | 0.8 | 83.7 | 15.9 | 0.4 | 95.5 | 3.5 | 1.0 |
| 2015 | 97.0 | 3.0 | 94.9 | 5.1 | 98.9 | 1.1 | 87.0 | 9.8 | 3.2 | 79.8 | 14.5 | 5.7 | 93.7 | 5.4 | 0.9 |
| 2016 | 94.7 | 5.3 | 88.0 | 12.0 | 98.1 | 1.9 | 88.0 | 7.6 | 4.4 | 70.5 | 17.1 | 12.4 | 96.7 | 2.7 | 0.6 |
| 2017 | 99.1 | 0.9 | 97.4 | 2.6 | 100.0 | 0 | 92.0 | 7.0 | 1.0 | 85.8 | 11.6 | 2.6 | 93.2 | 4.6 | 2.2 |
| 2018 | 96.2 | 3.8 | 96.7 | 3.3 | 95.8 | 4.2 | 89.5 | 5.5 | 5.0 | 80.8 | 7.2 | 12.0 | 95.8 | 4.2 | 0 |
| **Z value** | -22.1 |  | -17.4 |  | -18.9 |  | -9.3 |  |  | -7.2 |  |  | -11.7 |  |  |
| ***P*** _trend_ | **< .001** |  | **< .001** |  | **< .001** |  | **< .001** |  |  | **< .001** |  |  | **< .001** |  |  |

Note: NHFs, nursing home facilities.

# **Supplementary Table 4**. Results of Sensitivity Analysis

| **Characteristics** | **Dy/dx** | **95% CI** | | ***P* value** |
| --- | --- | --- | --- | --- |
| **Rurality (Ref: Urban)** |  | **Lower** | **Upper** |  |
| Rural | -82 | -124 | -41 | **< .001** |
| **Primary Caregivers (Ref: Informal)** |  |  |  |  |
| Formal | 82 | -71 | 235 | 0.29 |
| **Place of death (Ref: Home)** |  |  |  |  |
| Hospital | 666 | 524 | 808 | **< .001** |
| Nursing home facilities | -49 | -210 | 112 | 0.55 |
| **Age at death (Ref: 80-89)** |  |  |  |  |
| 90-99 | -199 | -263 | -135 | **< .001** |
| ≥100 | -278 | -343 | -214 | **< .001** |
| **Per capita household income annually (Ref: <** 391**)** |  |  |  |  |
| 391-942 | 57 | 15 | 99 | **0.01** |
| 942-3,060 | 130 | 83 | 177 | **< .001** |
| > 3,060 | 344 | 280 | 408 | **< .001** |
| **Main financial source** |  |  |  |  |
| **Retirement wage (Ref: No)** | 303 | 171 | 435 | **< .001** |
| **Timely medical services (Ref: Yes)** |  |  |  |  |
| No | -328 | -378 | -277 | **< .001** |
| Was not ill | -422 | -455 | -389 | **< .001** |
| **Disabilities in ADLs (Ref: No)** | 372 | 269 | 474 |  |
| **No of Comorbidities (Ref: 0)** |  |  |  |  |
| 1 | 88 | 46 | 131 | **< .001** |
| 2 | 210 | 146 | 274 | **< .001** |
| ≥ 3 | 232 | 170 | 294 | **< .001** |
| **Region (Ref: Eastern)** |  |  |  |  |
| Central | -165 | -214 | -116 | **< .001** |
| Western | -144 | -191 | -96 | **< .001** |
| Northeast | -107 | -182 | -32 | **0.005** |

Note: Dy/dx, Average marginal effect; CI, Confidence interval. ADL, activities of daily living. Limited to space required, only predictors of statistically significance were presented; and results of marginal association between gender, ethnicity, marital status, years of schooling, number of children even born, Family support as main financial source, white-collar jobs before retirement, living arrangement during the last year of life, self-rated health status, health behaviors and end-of-life medical expenditures were not presented. All the currency was presented in the US dollar.

# **Supplementary Table 5. Marginal Differences of Included Characteristics on Medical Expenditure during the Last Year of Life among Urban Deceased**

| **Characteristics** | **Dy/dx** | **95% CI** | | ***P* value** |
| --- | --- | --- | --- | --- |
| **Primary Caregivers (Ref: Informal)** |  |  |  |  |
| Formal | 620 | -429 | 1,669 | 0.25 |
| **Place of death (Ref: Home)** |  |  |  |  |
| Hospital | 967 | 578 | 1,357 | **< .001** |
| Nursing home facilities | -553 | -1,559 | 453 | 0.28 |
| **Age at death (Ref: 80-89)** |  |  |  |  |
| 90-99 | -383 | -569 | -197 | **< .001** |
| ≥100 | -633 | -883 | -384 | **< .001** |
| **Gender (Ref: Male)** |  |  |  |  |
| Female | -137 | -401 | 128 | 0.31 |
| **Ethnicity (Ref: Han)** |  |  |  |  |
| Minority | 154 | -266 | 574 | 0.47 |
| **Being Married (Ref: No)** | -108 | -419 | 203 | 0.50 |
| **Years of schooling (Ref: 0)** |  |  |  |  |
| 1-6 | 362 | 95 | 630 | **0.01** |
| ≥ 7 | 54 | -429 | 536 | 0.83 |
| **Number of children even born (Ref: 0-2)** |  |  |  |  |
| 3-4 | -40 | -383 | 302 | 0.82 |
| 5-6 | 57 | -281 | 394 | 0.74 |
| ≥ 7 | 206 | -136 | 548 | 0.24 |
| **Per capita household income annually (Ref: < 391)** |  |  |  |  |
| 391-942 | -32 | -264 | 199 | 0.78 |
| 942-3,060 | 236 | -136 | 608 | 0.21 |
| > 3,060 | 886 | 434 | 1,338 | **< .001** |
| **Main financial source** |  |  |  |  |
| **Retirement wage (Ref: No)** | 528 | 99 | 957 | **0.02** |
| **Family support (Ref: No)** | 271 | -120 | 662 | 0.18 |
| **White-collar jobs before retirement (Ref: No)** | 357 | -177 | 891 | 0.19 |
| ***Living arrangement in the last year of life*** |  |  |  |  |
| living alone (Ref: No) | -181 | -482 | 119 | 0.24 |
| living in the nursing homes (Ref: No) | -277 | -1,236 | 683 | 0.57 |
| living with spouse only (Ref: No) | 211 | -232 | 654 | 0.35 |
| **Timely medical services (Ref: Yes)** |  |  |  |  |
| No | -413 | -630 | -197 | **< .001** |
| Was not ill | -579 | -841 | -318 | **< .001** |
| **Self-rated health status (Ref: Very good or good)** |  |  |  |  |
| So so | 95 | -227 | 417 | 0.56 |
| Bad or very bad | -89 | -386 | 208 | 0.56 |
| Not able to answer | -190 | -504 | 124 | 0.24 |
| **Any disability in ADLs (Ref: No)** | 460 | 259 | 662 | **< .001** |
| **Number of Comorbidities (Ref: 0)** |  |  |  |  |
| 1 | -47 | -353 | 260 | 0.77 |
| 2 | 293 | -79 | 666 | 0.12 |
| ≥ 3 | 89 | -295 | 474 | 0.65 |
| **Bedridden before dying (Ref: No)** | 280 | 53 | 506 | **0.02** |
| ***Health Behavior*** ^a^ |  |  |  |  |
| **Physical exercises (Ref: No)** | -3 | -305 | 298 | 0.98 |
| **Smoking (Ref: No)** | 218 | -289 | 725 | 0.40 |
| **Drinking (Ref: No)** | -235 | -566 | 95 | 0.16 |
| **Region (Ref: Eastern)** |  |  |  |  |
| Central | -214 | -494 | 66 | 0.14 |
| Western | -269 | -531 | -7 | **0.045** |
| Northeast | -36 | -512 | 440 | 0.88 |

Note: Dy/dx, Average marginal effect; CI, Confidence interval. ADL, activities of daily living. a, self-reported in the previous survey. All the currency was presented in the US dollar.

# **Supplementary Table 6**. Marginal Differences of Included Characteristics on Medical Expenditure during the Last Year of Life among Rural Deceased

| **Characteristics** | **Dy/dx** | **95% CI** | | ***P* value** |
| --- | --- | --- | --- | --- |
| **Primary Caregivers (Ref: Informal)** |  |  |  |  |
| Formal | -208 | -798 | 382 | 0.49 |
| **Place of death (Ref: Home)** |  |  |  |  |
| Hospital | 594 | 243 | 944 | **0.001** |
| Nursing home facilities | -312 | -987 | 363 | 0.37 |
| **Age at death (Ref: 80-89)** |  |  |  |  |
| 90-99 | -88 | -195 | 20 | 0.11 |
| ≥100 | -296 | -426 | -167 | **< .001** |
| **Gender (Ref: Male)** |  |  |  |  |
| Female | -122 | -256 | 12 | 0.08 |
| **Ethnicity (Ref: Han)** |  |  |  |  |
| Minority | -48 | -222 | 126 | 0.59 |
| **Being Married (Ref: No)** | -24 | -242 | 193 | 0.83 |
| **Years of schooling (Ref: 0)** |  |  |  |  |
| 1-6 | 78 | -51 | 207 | 0.24 |
| ≥ 7 | 856 | -285 | 1,997 | 0.14 |
| **Number of children even born (Ref: 0-2)** |  |  |  |  |
| 3-4 | -36 | -226 | 153 | 0.71 |
| 5-6 | 82 | -121 | 285 | 0.43 |
| ≥ 7 | 157 | -42 | 356 | 0.12 |
| **Per capita household income annually (Ref: <** 391**)** |  |  |  |  |
| 391-942 | 42 | -99 | 182 | 0.56 |
| 942-3,060 | 198 | -87 | 483 | 0.17 |
| > 3,060 | 597 | 307 | 888 | **< .001** |
| **Main financial source** |  |  |  |  |
| **Retirement wage (Ref: No)** | 658 | -223 | 1,539 | 0.14 |
| **Family support (Ref: No)** | -348 | -711 | 15 | 0.06 |
| **White-collar jobs before retirement (Ref: No)** | -167 | -936 | 603 | 0.67 |
| ***Living arrangement in the last year of life*** |  |  |  |  |
| living alone (a) (Ref: No) | -51 | -214 | 111 | 0.54 |
| living in the nursing homes (Ref: No) | 25 | -970 | 1,020 | 0.96 |
| living with spouse only (Ref: No) | -40 | -303 | 224 | 0.77 |
| **Timely medical services (Ref: Yes)** |  |  |  |  |
| No | -281 | -408 | -155 | **< .001** |
| Was not ill | -231 | -369 | -94 | **0.001** |
| **Self-rated health status (Ref: Very good or good)** |  |  |  |  |
| So so | 122 | -22 | 267 | 0.10 |
| Bad or very bad | 188 | -29 | 405 | 0.09 |
| Not able to answer | -145 | -318 | 27 | 0.10 |
| **Any disability in ADLs (Ref: No)** | 246 | 70 | 422 | **0.006** |
| **Number of comorbidities (Ref: 0)** |  |  |  |  |
| 1 | 19 | -104 | 142 | 0.76 |
| 2 | 109 | -36 | 254 | 0.14 |
| ≥ 3 | 272 | 29 | 515 | **0.03** |
| **Bedridden before dying (Ref: No)** | 52 | -146 | 251 | 0.61 |
| ***Health Behavior* ^a^** |  |  |  |  |
| **Physical exercises (Ref: No)** | 3 | -142 | 149 | 0.97 |
| **Smoking (Ref: No)** | -71 | -242 | 100 | 0.42 |
| **Drinking (Ref: No)** | -49 | -200 | 101 | 0.52 |
| **Region (Ref: Eastern)** |  |  |  |  |
| Central | -254 | -433 | -74 | **0.006** |
| Western | -243 | -399 | -87 | **0.002** |
| Northeast | -230 | -491 | 31 | 0.08 |

Note: Dy/dx, Average marginal effect; CI, Confidence interval. ADLs, activities of daily living. a, self-reported in the previous survey. All the currency was presented in the US dollar.

# **Supplement Table 7. Comparison of Individual-level Characteristics between Deceased Reported and not Reported Medical Expenditure during the Last Year of Life**

| **Characteristic** | **All decedents (N=25,940)** | **Number (%) of Deceased Reported Medical Expenditure Data**  **(N= 24,050)** | **Number (%) of Deceased not Reported Medical Expenditure Data (N=1,890)** | **chi^2^** | ***P* value** |
| --- | --- | --- | --- | --- | --- |
| ***Rurality*** |  |  |  | 3.8 | 0.05 |
| Urban | 10,822 (33.9) | 9,900 (38.9) | 922 (33.5) |  |  |
| Rural | 15,118 (66.1) | 14,150 (61.1) | 968 (66.5) |  |  |
| ***Primary Caregivers*** |  |  |  | 45.3 | **< .001** |
| Informal | 21,909 (87.6) | 23,377 (69.2) | 1,468 (89.1) |  |  |
| Formal | 1,029 (6.8) | 1,229 (13.6) | 200 (6.2) |  |  |
| Missing | 1,112 (5.6) | 1,334 (17.2) | 222 (4.7) |  |  |
| ***Place of death*** |  |  |  | 39.7 | **< .001** |
| Home | 22,784 (84.0) | 21,336 (69.6) | 1,448 (85.2) |  |  |
| Hospital | 1,917 (9.0) | 1,703 (9.9) | 214 (8.9) |  |  |
| Nursing home facilities | 877 (5.0) | 748 (9.1) | 129 (4.7) |  |  |
| Missing | 362 (2.0) | 263 (11.4) | 99 (1.2) |  |  |
| **Age at death** | 97 (91,102) | 97 (91,102) | 97 (91,102) | 0.1 | 0.81 |
| 80-89 | 4,928 (77.7) | 4,541 (77.0) | 387 (77.8) | 23.2 | **< .001** |
| 90-99 | 10,524 (20.9) | 9,786 (19.1) | 738 (21.1) |  |  |
| ≥100 | 10,260 (0.8) | 9,543 (0.7) | 717 (0.8) |  |  |
| Missing | 228 (0.7) | 180 (3.2) | 48 (0.3) |  |  |
| **Gender** |  |  |  | 0.02 | 0.88 |
| Male | 10,109 (42.5) | 9,340 (42.9) | 769 (42.5) |  |  |
| Female | 15,831 (57.5) | 14,710 (57.1) | 1,121 (57.5) |  |  |
| **Ethnicity** |  |  |  | 0.9 | 0.36 |
| Han | 23,929 (92.1) | 22,151 (92.1) | 1,778 (92.1) |  |  |
| Minority | 1,712 (7.2) | 1,611 (7.7) | 101 (7.2) |  |  |
| Missing | 299 (0.7) | 288 (0.2) | 11 (0.7) |  |  |
| **Years of schooling** |  |  |  | 1.04 | 0.37 |
| 0 | 18,509 (65.8) | 17,250 (63.0) | 1,259 (66.1) |  |  |
| 1-6 | 5,782 (27.3) | 5,325 (30.3) | 457 (27.0) |  |  |
| ≥ 7 | 1,256 (5.4) | 1,125 (4.7) | 131 (5.5) |  |  |
| Missing | 393 (1.5) | 350 (2.0) | 43 (1.4) |  |  |
| **Number of children even born** |  |  |  | 1.4 | 0.25 |
| 0-2 | 5,460 (21.5) | 4,973 (26.5) | 487 (21.1) |  |  |
| 3-4 | 7,400 (28.4) | 6,872 (27.5) | 528 (28.4) |  |  |
| 5-6 | 7,229 (27.9) | 6,722 (23.2) | 507 (28.3) |  |  |
| ≥ 7 | 5,339 (20.7) | 5,009 (21.1) | 330 (20.6) |  |  |
| Missing | 512 (1.5) | 474 (1.7) | 38 (1.6) |  |  |
| **Per capita household income annually** |  |  |  | 153.1 | **< .001** |
| **<** 391 | 5,761 (31.2) | 5,543 (15.9) | 218 (32.6) |  |  |
| 391-942 | 5,789 (26.1) | 5,622 (10.3) | 167 (27.4) |  |  |
| 942-3,060 | 5,683 (17.8) | 5,504 (7.0) | 179 (18.7) |  |  |
| > 3,060 | 5,532 (13.8) | 5,363 (7.3) | 169 (14.3) |  |  |
| don't know | 1,566 (6.6) | 880 (33.4) | 686 (4.3) |  |  |
| Missing | 994 (4.7) | 546 (26.1) | 448 (2.7) |  |  |
| **Main financial source before dying** |  |  |  |  |  |
| **Retirement wage** |  |  |  | 137.3 | **< .001** |
| Yes | 2,581 (11.3) | 2,337 (12.7) | 244 (11.2) |  |  |
| No | 23,277 (88.0) | 21,709 (79.1) | 1,568 (88.8) |  |  |
| Missing | 82 (0.7) | 4 (8.2) | 78 (0) |  |  |
| **Family** |  |  |  | 133.5 | **< .001** |
| Yes | 21,143 (79.3) | 19,795 (64.5) | 1,348 (80.6) |  |  |
| No | 4,715 (20.0) | 4,251 (27.3) | 464 (19.4) |  |  |
| Missing | 82 (0.7) | 4 (8.2) | 78 (0) |  |  |
| **White-collar jobs before retirement** |  |  |  | 3.3 | **0.049** |
| Yes | 1,174 (4.9) | 1,032 (6.7) | 142 (4.8) |  |  |
| No | 24,701 (94.9) | 22,958 (93.2) | 1,743 (95.1) |  |  |
| Missing | 65 (0.2) | 60 (0.1) | 5 (0.1) |  |  |
| ***Living arrangement*** |  |  |  |  |  |
| **living alone** |  |  |  | 56.7 | **< .001** |
| Yes | 23,251 (88.2) | 21,734 (79.0) | 1,517 (89.0) |  |  |
| No | 2,430 (11.0) | 2,138 (15.5) | 292 (10.6) |  |  |
| Missing | 259 (0.8) | 178 (5.5) | 81 (0.4) |  |  |
| **living in the nursing home** |  |  |  | 48.4 | **< .001** |
| Yes | 645 (5.7) | 525 (13.1) | 120 (5.0) |  |  |
| No | 25,036 (93.6) | 23,347 (81.3) | 1,689 (94.6) |  |  |
| Missing | 259 (0.7) | 178 (5.6) | 81 (0.4) |  |  |
| **timely medical services** |  |  |  | 30.7 | **< .001** |
| Yes | 19,794 (81.9) | 18,444 (72.2) | 1,350 (82.7) |  |  |
| No | 4,847 (11.5) | 4,553 (9.7) | 294 (11.6) |  |  |
| Was not ill | 1,299 (6.6) | 1,053 (18.1) | 246 (5.7) |  |  |
| ***Health Condition*** |  |  |  |  |  |
| **Self-rated health status** |  |  |  | 0.6 | 0.59 |
| Very good or good | 9,536 (41.2) | 8,842 (39.0) | 694 (41.4) |  |  |
| So so | 7,891 (32.7) | 7,324 (35.5) | 567 (32.4) |  |  |
| Bad or very bad | 4,124 (19.6) | 3,798 (20.3) | 326 (19.5) |  |  |
| Not able to answer | 4,332 (6.4) | 4,034 (5.1) | 298 (6.6) |  |  |
| Missing | 57 (0.1) | 52 (0.1) | 5 (0.1) |  |  |
| **Any disability in ADLs** |  |  |  | 71.9 | **< .001** |
| Yes | 20,329 (69.8) | 18,980 (53.6) | 1,349 (71.2) |  |  |
| No | 5,051 (28.2) | 4,669 (34.6) | 382 (27.7) |  |  |
| Missing | 560 (2.0) | 401 (11.8) | 159 (1.1) |  |  |
| **Bedridden before dying** |  |  |  | 228.0 | **< .001** |
| Yes | 18,586 (69.2) | 17,320 (58.3) | 1,266 (70.2) |  |  |
| No | 7265 (30.1) | 6,727 (33.5) | 538 (29.8) |  |  |
| Missing | 89 (0.7) | 3 (8.2) | 86 (0) |  |  |
| **No of comorbidities** |  |  |  | 0.1 | **0.96** |
| 0 | 9,233 (32.6) | 8,634 (31.5) | 599 (32.7) |  |  |
| 1 | 8,258 (32.9) | 7,684 (33.4) | 574 (32.9) |  |  |
| 2 | 3,793 (15.2) | 3,475 (16.2) | 318 (15.2) |  |  |
| ≥ 3 | 4,656 (19.3) | 4,257 (18.9) | 399 (19.2) |  |  |
| ***Health Behavior*** ^a^ |  |  |  |  |  |
| **Physical exercise** |  |  |  | 0.4 | 0.52 |
| Yes | 5,057 (24.6) | 4,666 (26.3) | 391 (24.4) |  |  |
| **No** | 20,862 (75.4) | 19,366 (73.7) | 1,496 (75.6) |  |  |
| Missing | 21 (0) | 18 (0) | 3 (0) |  |  |
| **Smoking** |  |  |  | 349.4 | **< .001** |
| Yes | 22,251 (81.1) | 20,664 (78.5) | 1,587 (81.3) |  |  |
| No | 3,595 (18.2) | 3,378 (12.9) | 217 (18.7) |  |  |
| Missing | 94 (0.7) | 8 (8.6) | 86 (0) |  |  |
| **Drinking** |  |  |  | 79.7 | **< .001** |
| Yes | 4,098 (15.6) | 3,841 (14.1) | 257 (15.8) |  |  |
| No | 21,720 (83.6) | 20,181 (77.2) | 1,539 (84.1) |  |  |
| Missing | 122 (0.8) | 28 (8.7) | 94 (0.1) |  |  |
| **Region** |  |  |  | 0.6 | 0.61 |
| Eastern | 10,295 (36.0) | 9,397 (36.1) | 898 (35.9) |  |  |
| Central | 5,943 (25.2) | 5,619 (27.1) | 324 (25.1) |  |  |
| Western | 7,816 (31.0) | 7,247 (31.0) | 569 (31.0) |  |  |
| Northeast | 1,886 (7.8) | 1,787 (5.8) | 99 (8.0) |  |  |

Note: ADLs, activities of daily living. we grouped the 25,940 deceased as the initial dataset into deceased have any values on the EOL medical expenditures and those did not after dropping those aged less than 80 (N=1,939) or more than 106 (N=681) in the initial dataset of 28,560 deceased. Values were represented as No. (percentages) unless otherwise indicated. No, was calculated from study samples (unweighted). Percentages were calculated using the age-sex-rural/urban-specific sample weights. a, self-reported in the previous survey.
